# The Oral-Microbiome-Brain Axis and Neuropsychiatric Disorders: An Anthropological Perspective

**DOI:** 10.3389/fpsyt.2022.810008

**Published:** 2022-03-30

**Authors:** Grace B. Bowland, Laura S. Weyrich

**Affiliations:** ^1^Department of Anthropology, Pennsylvania State University, University Park, PA, United States; ^2^Huck Institutes of the Life Sciences, Pennsylvania State University, University Park, PA, United States

**Keywords:** oral microbiota, oral microbiome, dentistry, psychiatric disease, mental health

## Abstract

In the 21st century, neuropsychiatric disorders (NPDs) are on the rise, yet the causal mechanisms behind this global epidemic remain poorly understood. A key to these unknowns may lie within the vast communities of bacteria, fungi, and viruses in the body (microbiota), which are intimately linked with health and disease. NPDs were recently shown to be connected to gut microbiota, which can communicate with and influence the brain through the Gut-Brain-Axis (GBA). Parallel studies examining oral microbiota and their connections to the brain also suggest that microbes in the mouth can similarly influence NPD outcomes. However, the mechanisms and pathways that illuminate how oral microbiota and brain communicate in NPDs remain unknown. Here, we review identified mechanisms and pathways that oral microbiota use to engage the brain, and we lay the theoretical foundation for an oral-microbiota-brain axis (OMBA). Specifically, we examine established neuroinflammatory and immune system activation responses that underpin interactions between the oral microbiota and the central nervous system (CNS), detailing four specific mechanisms: (1) microbial and metabolite escape, (2) neuroinflammation, (3) CNS signaling, and (4) response to neurohormones. We then scrutinize why including the OMBA, in addition to the GBA, is critically needed to elucidate specific causal relationships between microbial dysbiosis and observed NPD development and progression. Furthermore, we argue for comprehensive, interdisciplinary approaches that integrate lab-based microbiome research and population-level studies that examine the OMBA to improve NPDs. We specifically identify key anthropological perspectives that integrate sociocultural, epidemiological, genetic, and environmental factors that shape the oral microbiome and its interactions with NPDs. Together, future studies of the OMBA in conjunction with interdisciplinary approaches can be used to identify NPD risks and improve outcomes, as well as develop novel intervention and treatment strategies.

## Introduction

Microbiome research has been driven by new revelations regarding the complex interactions which contribute to human health and disease, particularly in the global rise of neuropsychiatric disorders (NPDs) and non-communicable diseases (NCDs) (1). Mental health has long been a confounding subject for researchers and practitioners of health and medicine, and breakthroughs in treatment and preventative measures are slow. This is largely due to the lack of understanding regarding the causal factors of neurophysiological and psychiatric disorders and is compounded by the complex physiological, genetic, social, and environmental factors that each contribute to mental and neurological health (2).

Increasingly, researchers and health experts recognize that no one factor, or microbe, can be exclusively to blame in NCD or NPD development, but recent findings have shown that the brain is much more connected to systemic health than previously believed. For example, the microbial communities within the body (microbiota) can play an integral role in NCDs, such as obesity and diabetes, and affect mental and neurological health (1–4). This research has predominantly focused on the GI tract, which contains most of the microbiota associated with the human body, both in numbers and species diversity (5). As a result, the primary findings in the context of microbe-brain interactions are facilitated through the Gut-Brain-Axis (GBA). The GBA refers to the complex bidirectional interactions and processes utilized by the gut microbiome and brain to communicate with one another (6). Broadly, the purpose of the GBA is to integrate gut functions with the brain’s emotional and cognitive centers. This is achieved through numerous mechanisms, including immune activation, entero-endocrine signaling, intestinal permeability, and enteric reflex (6, 7). Interest in the GBA and its connections to human health and behavior has led to an emerging field of study known as microbial endocrinology, which has sought to elucidate the functional pathways through which gut microbiota engage in the GBA (1, 2, 8–10). The primary pathway that allows interactions between the central nervous system and gut is primarily facilitated by hormones and mediated by the long branching vagus nerve (11).

As bacteria possess hormone receptors and perform and utilize quorum sensing, they can sense and produce autoinducer (AI) molecules, which function within bacteria in a similar manner to hormones in the host by regulating functions of bacterial growth, motility, and virulence (11, 12). It was further discovered that these AI molecules can also modulate host cell transduction and engage in crosstalk with host hormones which activate signaling pathways within the host organism (11, 13). The ability of AI molecules and hormones to interact in a similar fashion with both host and microbe forms the foundation of their communication and influence on one another. Commensal bacteria in the gut directly act on host hormones by producing and secreting hormones linked to host metabolism, immunity, and behavior ((7), and (11)). For example, a study by Iyer et al. (14) found that gut microbiota can influence the central nervous system (CNS) through production of hormones and other related molecules, such as serotonin, acetylcholine, histamine, and melatonin, which act as local neurotransmitters. Bacterial neuro-immuno-endocrine mediators are also involved in the breakdown of dietary components (15), immune system education, human development (16–18), and toxin degradation (11). As the microbe can modulate the host through AI, the host is also able to influence and alter gut microbiota through hormone production. A study conducted by Mudd et al. (19) reported catecholamines can modulate bacterial gene expression, with serum cortisol mediating the relationship between fecal *Ruminococcus* and brain N-acetylaspartate (NAA). The results indicated that the prescence of *Ruminococcus* was negatively associated with serum cortisol levels and brain NAA, a biomarker for neurological health. This can in turn affect the health of the host.

Microbial endocrinology and GBA research lay an important foundation for understanding functional processes of microbe–CNS interactions. This communication pathway between host and microbiome is not likely unique to the gut microbiome alone. It has been noted that functional pathways are conserved across microbiome sites and species composition of communities (16). Given that some microbes are tightly co-evolved with their hosts standpoint, it is logical that the microbiome can be in constant communication with the host’s neurophysiological system (20). Furthermore, the ability of bacteria to synthesize and recognize the same neurotransmitters that are found in the host suggests a bidirectional environment where the microbiome can influence the host and the host influence the microbiome. This communication between host and microbiome is mediated by a commonly shared evolutionary pathway of intercellular signaling (20). This once again indicated that host systems and microbial communities are not isolated from one another and supports the argument that there are tangible physiological pathways affecting mental health and illness associated with microbiota.

Although the GBA research has rapidly enhanced our understanding of microbe and CNS interactions, this research often focuses only on the lower digestive tract and neglects another important environment: the oral microbiome. The mouth is the beginning of the digestive system and a primary entry point for all things (microbial and otherwise) to access the internal body. As with the gut, oral microbiome research is shifting to perspectives focused on holistic, systems-level understandings of its functions and interactions with our bodies (21–24). Recent findings have shown that oral microbiome health is not only an indicator of oral health problems, such as caries and periodontal disease, but is also a key player in systemic disease, including obesity, diabetes, and neurological and psychiatric disorders (25–28). Indeed, oral microbial communities are likely involved in the same complex, bidirectional crosstalk between the brain and central nervous system as the gut microbiome. Although the oral microbiome has been comparatively less researched than the gut, current published literature shows promising complementary results to gut microbiome research, which points to similar neuro-immuno-chemical processes in host-oral microbe interactions.

The microbial diversity of bacteria, fungi, and viruses present in the mouth is second only to that of the gut and has led to more questions regarding the importance of these diverse and complex microbial networks in host health and development (29). There is established recognition among the medical, dental, and periodontal research fields that the oral microbiome plays an important role in systemic health (30). For example, disruption of the oral microbiome is a contributing factor to several chronic diseases across the body, including endocarditis, rheumatoid arthritis, and osteoporosis (31–34). The extent of oral microbial importance to health goes beyond the potential for bacterial pathogenesis infection. Oral health, gum disease, and periodontitis have been found to play a direct role in the development and progression of non-communicable diseases (NCDs), such as obesity (35), diabetes (36), and a number of cancers, including colorectal and esophageal cancer (37). With these insights, attention on the oral microbiome and its corollary relationship to systemic disease has focused on its exciting potential for early diagnostic purposes and intervention of NCDs. For example, Willis and Gabaldón (37) suggest that the presence of *Lactobacillus* in the mouth is associated with dental carries, and therefore, it could be used as an ancillary indicator of poor oral hygiene, which has a strong relationship to cancer development. In another example, Xiao et al. (38) notes the Craig et al. (35) study, where the ratio of Firmicutes-to-Bacteroidetes in the oral microbiota in children was strongly associated with weight gain. They conclude that the oral microbiome could be a valuable tool in assessing obesity risk. However, Xiao et al. (38) also concedes that we have yet to determine the direct mechanism underlying this relationship.

The results of these studies demonstrate that oral microbiota are directly involved in NPDs, and as with NCDs, exploration into diagnostic and intervention and treatment purposes is necessary. Current literature has posited the potential meanings for associations between certain oral species and NPDs, but there is yet to be a comprehensive attempt at compiling the direct mechanisms by which the oral microbiota affect the host CNS, and thereby cause or contribute to NPDs. This is a critical step in bringing the suggested benefits of oral microbiome research for health into actual medical practice.

**FIGURE 1:**
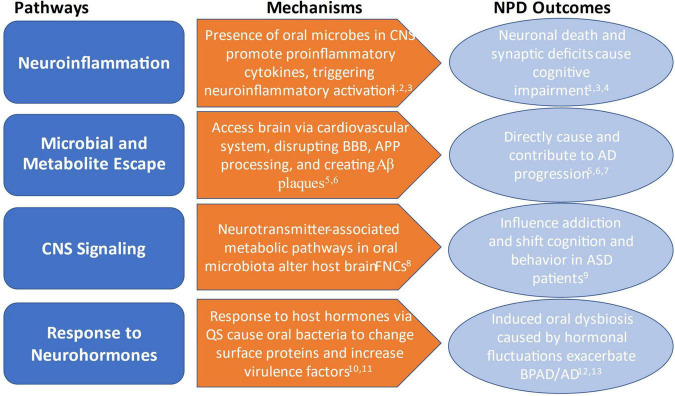
Overview of mechanisms by which oral microbiota influence and contribute to NPD outcomes. ^1^Xue et al. (25); ^2^Xia et al. (43); ^3^Dinarello et al. (94); ^4^Ding et al. (45); ^5^Bulgart et al. (39); ^6^Watanabe et al. (40); ^7^Dominy et al. (42); ^8^Lin et al. (26); ^9^Olsen and Hicks (50); ^10^Jentsch et al. (56); ^11^Duran-Pinedo et al. (30); ^12^Cunha et al. (28); ^13^Vieira et al. (33).

Where previous reviews have focused on the indirect implications of oral microbial research in assessing risk for NPD and NCD development, this review will examine new research illuminating the direct causal mechanisms by which oral microbiota contribute to disease development and progression. Drawing on these studies, we propose a theoretical basis for an oral-microbiota-brain axis (OMBA): (1) microbial and metabolite escape, (2) neuroinflammation, (3) CNS signaling, and (4) response to neurohormones (Figure 1). In addition, we explore how applying this OMBA framework can help to connect microbial dysbiosis to NPD progression and outcome. Lastly, we will also argue that OMBA research needs to move beyond studies in microbiology, psychiatric, and nutritional health sciences and include anthropological methods and approaches to the study of the oral microbiome and mental health and discuss some potential oral microbiome-based solutions with implications for NPD treatment. For a summary highlighting the key studies and findings discussed in this review, please see Table 1.

**TABLE 1:** Summary of key studies and findings discussed in this review.

Study	Key findings	Mouse Model (yes or no)
Cunha et al. (28)	Identify increased risk of CP in participants with BAPD, and associations between periodontitis and total oral bacterial load with the depressive phase of BPAD	No
Ding et al. (45)	Suggest *P. gingivalis* periodontal infection causes cognitive impairment via release of pro-inflammatory cytokines	Yes
Dominy et al. (42)	Establish direct pathway by which oral microbe *P. gingivalis* influences AD progression via disruption of APP processing	Yes
Duran-Pinedo et al. (30)	Cortisol induces shifts in oral microbiome gene expression consistent with those seen with periodontal disease and progression	No
Handsley-Davis et al. (69)	Differences in oral microbial profiles between Indigenous and non-Indigenous Australians highlights need for inclusion of Indigenous communities in oral microbiome research to generate specific knowledge about oral microbiota and chronic disease to address gaps in Indigenous health	No
Lassalle et al. (41)	Oral microbial profiles from hunter-gatherer and traditional farmers in the Philippines and Western populations suggest major shifts in diet selected for different microbial communities and helped shape modern oral pathogens	No
Liddicoat et al. (93)	Exposure to dust from high biodiversity soil changed gut microbiota and reduced anxious behavior in mice, suggesting microbiome supplementation via environment is important for physical and mental health	Yes
Lin et al. (26)	Pattern between smoking-induced oral microbiome dysbiosis and brain functional connectivity alternation, demonstrating direct involvement of oral microbiome in mediating brain activity	No
Mills et al. (91)	Co-benefits of interdisciplinary focus on microbiome biodiversity, including improved human health outcomes and investment opportunities for biodiversity conservation and restoration	No
Monteiro et al. (71)	Demonstrates the primary role played by parental disease in microbial colonization patterns in their children and the early acquisition of periodontitis-related species	No
Selway et al. (58)	Microbial diversity from nasal and skin samples of participants exposed to urban green spaces in three countries shows exposure to these spaces can increase microbial diversity and alter human microbiota composition	No
Watanabe et al. (40)	Establishes direct causal pathway between oral microbe *S. mutans* colonization of the brain and cognitive impairment via *S. mutans* expression of collagen-binding activity	Yes
Xue et al. (25)	Oral microbes associated with periodontitis disrupt intestinal and blood-brain barriers and induce cognitive impairment through increasing serum contents of proinflammatory cytokines and lipopolysaccharide (LPS) within the brain and body	Yes

## Microbial and Metabolite Escape and Disease

Many microbial species are site-specific in the host body, where they perform beneficial functions and are not typically associated with disease. However, when microbes escape from their niche and travel to other locations, they can cause disease elsewhere in the body by disrupting bioprocesses and secreting biproducts (e.g., metabolites), which can be toxic in some conditions. Microorganisms that colonize the body in the wrong spot can be a mechanism that underpins disease. This may be more critical in times of disease when oral microbial communities are ‘dysbiosed’ and certain potentially pathogenic microbes are in higher abundance.

Through the bloodstream, these escaped oral microbes and their metabolites travel to new regions of the body, where they can aggregate in different tissues causing diseases such as endocarditis and rheumatoid arthritis (31, 34). Oral microbiota can also make it to the brain via the cardiovascular system, where they are thought to be directly responsible for disruption of vital neurological functions and degradation of the brain through accumulation of toxic bioproducts (39). In mice, the collagen-binding activity of cnm-positive *Streptococcus mutans*, an oral microbe associated with dental carries, can leave the oral cavity in the blood and induce cerebral hemorrhaging by disrupting the blood–brain barrier (BBB). Endothelial cells of cerebral vessels are damaged during hemorrhage, exposing collagen on the surface of the blood vessel. If cnm-positive *S. mutans* is present in the bloodstream, its collagen binding activity can further cause hemorrhaging by adhering to and damaging the vascular endothelium in the brain (40). Similarly, the bacterium *Porphyromonas gingivalis* exists in many orally healthy people (41) but can be a key pathogen in the development and progression of periodontitis. However, *P. gingivalis* has been known to escape via the bloodstream and make its way to the brain, where it colonizes and releases neurotoxic proteases called gingipains. These metabolite gingipains are involved in disruption of the transmembrane protein Amyloid precursor protein (APP) processing, which is responsible for synaptic stability and neuronal growth and protection (39). This causes the improper cleaving of APP and creates amyloid beta (Aβ) protein plaques that in turn instigate neuronal death (39, 42).

The cascading affects caused by microbial and metabolite escape to the brain can directly result in disease. In individuals with Alzheimer’s Disease (AD), oral microbes, such as *P. gingivalis*, can directly affect disease progression and outcomes through the disruption of APP processing and subsequent Aβ accumulation (39, 42). AD is a progressive neurodegenerative disease demarcated by individuals that experiences both short- and long-term memory loss, mood swings, forgetfulness, and impaired decision making (39). Increased Aβ production leads to plaque buildup and increased the levels of phosphorylated tau, which is a protein essential for dendritic and axonal processes (39, 42). While oral microbial escape is now well appreciated, further research investigating which species are capable of escape and how they modify the CNS is critically needed, given the severe reactions associated with their escape in other diseases, such as cardiovascular disease.

## Neuroinflammation

The presence of oral microbes and their byproducts within the bloodstream and CNS can have detrimental effects on the brain by stimulating the body’s neuroinflammatory response. During this process, expression of inflammatory mediators such as pro-inflammatory cytokines are promoted (25), mounting a sometimes mild, but constant immune response. Systemic inflammation can induce alterations in neurovascular functions, resulting in an increase in the blood–brain barrier permeability, reduction of nutrients, and buildup of toxins within the brain (43).

Oral microbes associated with dysbiosis and oral disease play critical roles in the production of pro-inflammatory cytokines, both in localized tissues and systemically (25). Proinflammatory cytokines, such as interleukin 1 (IL-1) and tumor necrosis factor (TNF), are molecules that can intensify a host’s immune response and are responsible for producing fever, inflammation, and tissue destruction (44). *P. gingivalis* has been shown to cause the body to release pro-cytokines TNF-α, IL-6, and IL-1β, resulting in neuroinflammation (45) during chronic periodontal disease (CP). CP is a polymicrobial condition which causes dysbiosis within the oral microbiome and is responsible for immune system activation (25). In fact, CP has been additionally linked to increased bacterial lipopolysaccharide (LPS) levels in the brain, which indicate the presence of bacteria and disruption of the BBB. With elevated LPS levels, expression of the host receptor toll-like receptor 4 (TLR4) also increases, triggering neuroinflammatory activation (25).

Neuroinflammation is likely to play key roles in the relationships between oral microbes and the CNS. A key example is how neuroinflammation associates with cognitive impairment (CI). CI is a pathological state associated with marked cognitive and neurological decline (46). It has been surmised that CP-induced neuroinflammation causes neuronal loss and synaptic deficits. This is crucial, as neural and synapse health are vital in maintaining normal cognitive function (25). As CP is a polymicrobial disease, it is likely that more species, beyond *P. gingivalis*, can contribute to neuroinflammation. Further research is needed to identify these oral microbes, explore the microbial mechanisms used to trigger this immune response during CP, and understand the levels of activation required to influence the CNS.

Furthermore, oral microbial involvement in NPDs may also be linked with broader systemic inflammation. Investigations of oral microbial involvement in neuroinflammation has found that oral dysbiosis can disrupt the GBA, causing gut inflammation and further contributing to systemic inflammatory response in the host (25). The cascading effect of oral microbial dysbiosis on the GBA may explain why chronic inflammatory diseases have been shown to co-occur with NPDs. For example, research on Inflammatory Bowel Disease (IBD), a group of disorders involving chronic inflammation of the digestive tract, noted that individuals with IBD had worse oral health and higher risk of periodontitis than those who did not have IBD (47). Other studies of IBD have found increased incidence, and prevalence of psychiatric disorders such as depression, anxiety, and bipolar disorders have also been noted in the IBD population (48). Further research into the influence of oral microbiota in chronic inflammatory diseases and NPD outcomes is needed to this point.

## Central Nervous System Signaling

Recent findings have shed light on the ways that oral microbes can negatively influence neurological processes and shape cognition and behavior. However, oral microbial species may be vital to brain function, and therefore, altering microbial community composition could affect this by acting on specific neural pathways in the brain. Functional network connectivity (FNC) refers to the temporally dependent activation patterns of anatomically separate brain regions (49). These networks allow for different parts of the brain to communicate with one another and are responsible for functions such as cognitive-control, attention, and rapid information processing (26).

Analysis of FNC and oral microbial metabolic pathways in smokers identified enrichment of neurotransmitter-associated pathways in microbiota. These pathways include tyrosine metabolism and production of glutamate-glutamine and glutamatergic synapse. The production of neurotransmitters from the glutamine and glutamate pathway is stimulated by smoking and is involved in the reward circuit neural functions (26). In this way, oral microbiota could directly influence the reward pathways in the brain pertaining to smoking behavior and dependency, therefore altering the typical interactions between oral microbes and FNC (26). The FNC between networks involved in cognitive control and information processing were weaker in smokers, suggesting that oral microbiota may also play a role in dysregulating neurocognitive ability (26).

Breakthroughs in determining specific interactions between oral microbiota and brain circuit function are potentially valuable for studying psychiatric disorders as well. Research recently described an association between oral microbiota and Autism Spectrum Disorder (ASD). Research has indicated that children with ASD have lower oral bacterial diversity than their non-ASD peers, which is consistent with findings from the gut (50). The genera *Haemophilus* in saliva and *Streptococcus* in dental plaque were significantly more abundant in children with ASD, whereas *Prevotella*, *Selenomonas*, *Actinomyces*, *Porphyromonas*, and *Fusobacterium* were reduced. A depletion of the Prevotellaceae family co-occurrence network was also detected in plaque from ASD patients (50). This could point to the role of certain oral microbiota in exacerbating ASD symptoms, as ASD patients were noted to have increased abundances of bacteria associated with periodontal disease, immune activation, and inflammatory response. Further research utilizing study designs similar to that of Lin et al. (26) may illuminate causal factors of oral microbiota in shifting cognition and behavior in ASD patients. Downstream research using gnotobiotic mice or targeting the depletion of certain microbes in model systems may also provide further insights into how microbes contribute to routine brain function.

Another potential, yet understudied, mechanism by which oral microbiota may interact with the CNS is through afferent signaling via the vagus and trigeminal nerve complex. The vagus nerve runs from the brain to the abdomen and is responsible for relaying signals between the digestive tract and CNS. Research regarding gut microbiota interaction with the GBA has established that the vagus nerve is able to sense gut microbial metabolites via afferent fibers and transfer this information to the CNS (51). The trigeminal nerve is similarly responsible for innervating and relaying signals between the brain and oral and nasal cavities, and also contains connections to the vagus nerve via the main sensory nucleus (52). Therefore, it is likely that the same afferent signaling between gut microbes and the brain could very well be occurring within the mouth via the vagus and trigeminal nerve complex.

## Response to Neurohormones and Neuroendocrine Signaling

Host hormones signal to the host and simultaneously influence the oral microbiota. Much of this research centers on the hypothalamic pituitary adrenal (HPA) axis, which is primarily responsible for coordinating the body’s response to stress through the release of catecholamines, such as cortisol. Cortisol is a primary regulatory factor in numerous host systems, including immune response, host growth, and development (53).

A number of studies examined the interactions between the oral microbiome and cortisol and showed host modulation of oral bacterial gene expression through cortisol (54, 55). Through quorum sensing, bacteria may use catecholamine hormones as siderophores—molecules which bind and transport iron. As such, catecholamines may also cause bacteria to change outer surface proteins and increase cytotoxic activity and other virulence factors (56). Exposure to increased levels of cortisol have been shown to oral microbial composition and caused upregulation of virulence factors in certain microbial species involved in periodontitis and carries, including *Fusobacterium* species and *P. gingivalis*. These shifts in oral microbial community composition are associated with increased inflammation associated with gingivitis and periodontal disease (30, 56).

Microbial response to hormones may also play a part in exacerbating psychiatric disorders, such as bipolar affective personality disorder (BAPD) (28). Differences in oral microbiome composition were reported in individuals with BAPD compared to those without, and an association between increased risk of CP was identified in those with BAPD versus healthy individuals (28). In participants with BAPD and CP, the abundance of *Aggregatibacter actinomycetemcomitans* and *P. gingivalis* counts were significantly higher, and periodontitis was strongly associated with the total bacterial load and the depressive phase of BPAD, which also impacts catecholamine levels (28). This may suggest that CP induced by oral microbial response to shifts in hormones may exacerbate the symptoms of BPAD, and vice versa. In addition, PD may also be associated with neuroendocrine changes due to aging and cognitive impairment leading to AD. The primary factor responsible is thought to be an increase in cortisol with age, which induces oral microbial dysbiosis leading to PD (57). Women have an increased risk of developing PD and oral inflammation in old age (33), which may be due to decreases in estrogen (an important immune regulator) during menopause and increases in cortisol with aging (33). The immune deficits observed with lower estrogen, combined with increased cortisol levels, could be vital cues in changing oral microbial composition and inducing PD-associated dysbiosis. This may also be a responsible factor for why a large number of AD patients are female (33). However, much more research is needed to establish the functional mechanisms behind these relationships and explore how oral microbiota may respond to a plethora of different hormones.

## Anthropological Insights Into Microbiome Research With Implications for Neuropsychiatric Disorders

Establishing the direct functional pathways through which the human microbiome affects health outcomes, especially in the nervous system, is vital, but it is only one piece of the puzzle. As noted above, the oral microbiome is influenced by the host but is also shaped by our lifestyles (41), environments (58), diets (59), and exposures to toxins (26). Oral microbial dysbiosis associated with disease can be triggered by changes brought about through environmental disturbance and dietary shifts, leading to host disease (58, 60). Thus, understanding the human factors that shape our microbiome is likely to be an important mediator of NPD disease outcomes.

Currently, most microbiota studies, regardless of disease state, are from Industrialized people from the United States of America or China (61) and include data from Western, educated, industrialized, rich and democratic (WEIRD) populations (37). Despite making up less than 20% of the world population, “WEIRD” populations have historically accounted for 60–90% of subjects in psychological studies (62). As behavioral presentations of mental illness are heavily influenced and filtered through our sociocultural experiences, models linking oral microbiota and NPDs that are built using only a narrow range of participants are inadequate to fully understand and diagnose NPDs, nor understand how different oral microbiota compositions influence NPDs and other diseases (1, 63). While psychiatry has made a push to include models which incorporate culture as a factor which influences peoples’ experience of illness, there is still a need for theory that addresses the complex interactions between sociocultural and biological factors which produce NPDs across the diversity of human experiences (64). Anthropology can provide this missing theoretical framework to address this, and thus contribute to the perspective in current microbiome and mental health research, by situating lifestyle factors such as diet, behavior, and environment within the context of NPDs and the oral microbiome.

Anthropological perspectives allow researchers to disentangle the factors that shape microbial diversity and community composition of the oral microbiome (26, 41, 65), which in turn can influence NPDs. For example, NPDs are being investigated with particular focus on nutrition and diet (1, 10), but there is limited information on how this links to microbial shifts associated with lifeways (e.g., culture). Some of this research has been done using interdisciplinary methods between psychiatry and nutrition, dubbed “nutritional psychiatry,” as this field focuses on the influences of diet on the GBA as an integral factor in neuropsychiatric disorders (10); however, this work has excluded the OMBA, especially in the context of diverse lifeways lived by different Indigenous people globally. This is critical, as large-scale dietary differences have been shown to influence oral microbiota, as they do gut microbiota. For example, oral microbial samples from hunter gatherer and traditional farming communities in the Philippines revealed that, when compared to data from individuals living on a Western diet, the abundance of core microbial oral species were significantly correlated with subsistence strategy (41). Other examples have demonstrated that some Indigenous and traditional hunter-gatherer populations carry higher loads of potential oral pathogens that are linked to PD, such as *Prevotella*, *Porphyromonas, Treponema*, and *Tannerella*, than traditional farmers (41, 60, 66, 67), despite having less PD in those hunter-gatherer communities. As such, research examining intertwining dietary and nutritional differences in the context of human cultural, lifestyle, and environmental differences is needed to better understand the relationships between OMBA and NPDs.

Similarly, anthropology can help improve our understanding of OMBA and NPD linkages by examining how different histories and lifeways affect oral diseases that may differentially influence health, such as caries or periodontal disease. For example, although there is still limited research regarding oral microbial diversity among Indigenous groups, analyses of oral microbiota from Native American individuals indicated that the microbial communities of Indigenous participants significantly differed from their non-native counterparts, including high abundances of the genus *Prevotella*, which have been implicated in periodontal disease (68). In addition, it is also established that Aboriginal Australians and Torres Strait Islanders also suffer from higher incidences of NPDs and NCDs than their non-Indigenous counterparts (69). Recent research suggests that this health disparity may be complicated by the higher prevalence of oral disease, such as tooth decay and moderate to severe periodontal disease, experienced by Indigenous Australians in comparison to non-Indigenous Australians (69). These systemic differences were correlated with established differences microbial diversity and composition of Indigenous and non-Indigenous Australians, suggesting that these differences in oral microbiota-related diseases may be specific to Indigenous Australians via a unique oral microbiome (69). This oral microbial dysbiosis may be the result of evolutionary or sociocultural differences or, more likely, are reinforced by economic and social inequalities that result in greater burdens of disease. Higher densities of oral disease in certain populations or groups of people could result in higher incidences of downstream systemic diseases. While this observation shapes our understanding of how oral disease may be linked to mental health outcomes, this relationship between microbes and disease is likely to be unique across different human populations and therefore need an anthropological lens.

Anthropology also provides a structure by which to examine sociocultural factors effecting oral and mental health. For example, social and community interactions impact oral microbiome composition. Close family members and romantic partners possess similar microbial compositions to one another, likely facilitated by similar exposures as well as transferring of microbes via direct contact (70, 71). This is important to consider, as these could contribute to an individuals’ oral microbial composition and health. For example, evidence suggests that offspring of parents with periodontitis are preferentially colonized by bacteria known to be involved in periodontitis, including *P. gingivalis, A. actinomycetemcomitans, Streptococcus parasanguinis*, and *Fusobacterium nucleatum* (71). Furthermore, plaque control did not appear to change this, suggesting that interventions at the individual level through dental hygiene may not be adequate (71). Microbial inheritance from parent to child is an important aspect of periodontal disease, and thus may influence NPD risk and outcomes. As clinicians often place the emphasis on oral hygiene as the solution to slowing periodontitis, and now potentially NPDs, other contributing factors, such as inherited microbes, may not be within the individual’s control and need further mitigation.

Examination of sociocultural influences on the oral microbiome may also be extended to gender. Very recent studies have indicated significant differences in oral microbial community composition and abundances between adolescent girls and boys and elderly men and women (72, 73). However, it should be noted that these findings are likely confounded by biological factors of sex. Research has also found that sex and gender are major influencing factors in NPD presentation and outcome (74). This has led to a call for inclusion of gender and sex in a more tailored approach to NPD diagnosis and treatment (74). Therefore, it is important that such research be expanded to include the wider spectrum of gender identities in order to assess how the social constructs of gender may play a role in shaping the relationship between oral microbiota and NPDs.

**FIGURE 2:**
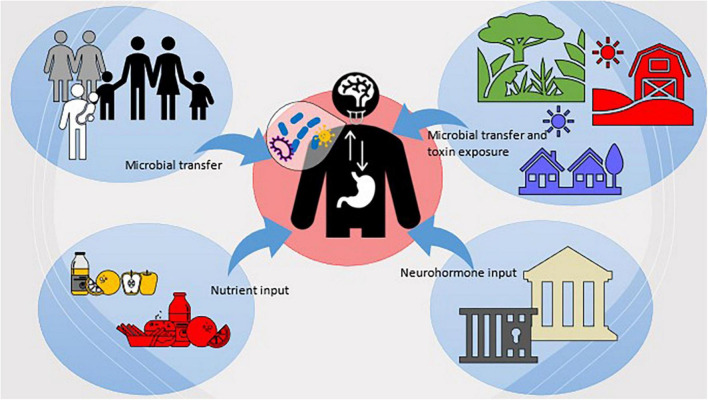
Visualization of the sociocultural and environmental factors which influence host microbiota and the OMBA.

Further to this, larger sociocultural influences, such as social and economic injustice that can increase periodontitis occurrence and exacerbate NPDs, can also be better understood with an anthropological lens. Anthropologists have drawn heavily on structural violence theory – a tripartite model of cultural violence, structural violence, and direct violence (75) that results from the institutionalization of cultural power structures and blocks access to care, opportunities, and resources, and marginalize specific populations—to identify and understand why some groups are disproportionately affected by oral diseases and vulnerable to NPDs. For example, Indigenous populations experience worse health, higher NCD and NPD burdens, and larger oral disease prevalence than non-Indigenous populations (69, 76). Studies examining the relative oral health disparities between Indigenous and non-Indigenous persons from New Zealand, Brazil, Australia, Canada, and the United States have found that Indigenous people consistently experience worse oral health than their non-Indigenous counterparts regardless of location (77, 78). Experiencing higher levels of structural violence could influence microbial exposure, colonization, and microbiome responses (i.e., microbiome’s response to stress hormones), which would ultimately alter systemic disease outcomes and therefore predispose Indigenous peoples to higher NCD and NPD burden. Further, early life stress may also contribute indirectly to NPD development later in life via the oral microbiome by shifting microbiota composition toward a disease-associated state (71, 78, 79). Without research and intervention efforts that examine sociocultural and environmental exposures across different settings, these health gaps will continue to exist. Figure 2, summarizes these concepts regarding the relationship between the OMBA and sociocultural and environmental factors.

## Solutions and Preventative Measures in Treating Neuropsychiatric Disorders Through the Oral Microbiota

Expanding our understanding of the environmental factors and microbial taxa associated with a healthy oral microbiome will open new avenues for intervention and prevention of NPDs. However, this knowledge should also underpin novel strategies to modulate or alter the microbiota to limit disease. Several tools already exist to manipulate or modulate oral microbiota, albeit with varied effectiveness, while new tools and pharmaceutical interventions are currently being developed. These single approaches or combined approaches may provide unique solutions or novel intervention therapies in how health practitioners think about preventing and addressing NPDs.

Antimicrobials are our first line of defense against pathogenic infection and work to eliminate specific species (i.e., phage treatment) or whole groups or families of microorganisms (i.e., antibiotics). Removing microbes found to be associated with NPDs may be a new way of addressing the symptoms or severity of NPDs in the future. However, antibiotics administered orally have been shown to have little impact on oral microbiota (80), suggesting that topical or direct application of antibiotics in the oral cavity may be more effective. Further, phage therapies designed against oral microbes have had little efficacy (i.e., against *F. nucleatum*). As such, encouraging growth of beneficial or potentially non-pathogenic microbes in the mouth may be a more appropriate way to modify the oral microbiota in support of addressing NPDs.

Probiotics have been increasing in popularity over the past decade as a form of health maintenance and disease prevention (81). Some research has indicated that *Lactobacillus* and *Bifidobacterium* spp. can aid in controlling the growth of cariogenic streptococci in the mouth (82). However, the effectiveness of probiotics often only lasts as long as the user is actively taking them, suggesting that the bacteria need additional factors to successfully colonize the oral cavity (83). Furthermore, most clinical trialing of these probiotics occurs within WEIRD populations, making it difficult to speak to their efficacy across populations. Identifying new microbial strains which contribute to oral health in individuals, as well as the environmental factors which dictate their success, will be vital in creating more beneficial probiotics to treat oral disease, and potentially act as preventatives for NPDs.

A novel therapy that seeks to address key holes in probiotic research is the implementation of microbial transplantations. Microbiota from a healthy donor are transplanted or seeded into the body of the recipient. While most research in microbial transplants has focused on the lower digestive tract, as fecal microbial transplants are successfully used to treat antibiotic-resistant *Clostridium difficile* infections (84), this technology is currently being developed in the mouth (61). Oral microbial transplants (OMTs) may be able to serve as a first line of defense against caries or be used as a treatment of periodontal disease, but this has not been tested in humans (85). In the future, applications for OMTs should be explored for their use in alleviating symptoms of systemic diseases, such as NPDs. Nevertheless, OMT therapies should account for evolutionary, environmental, and sociocultural factors which may affect transplant efficacy and utilize oral microbiota that have not been linked to NPDs.

Oral hygiene products, like toothpastes and mouthwashes, manage oral microbial communities by limiting growth of certain species and may be a way to promote colonization of desired oral microbes. For example, current toothpastes are formulated with chemicals with antimicrobial properties, such as fluoride, which has been shown to decrease overall microbial load and diversity (86) in addition to promoting healthy enamel. Further, many mouthwashes contain alcohol to kill microorganisms. While these are tools used in daily hygiene practices to prevent and reduce oral disease, they may also be tools to help modulate microbes that are linked to NPDs. New research is examining additional chemical compounds that may maintain oral hygiene without disrupting the commensal balance in microbiota (87). These compounds could also be examined in the context of treating or reducing NPDs symptoms in the future.

An important component to consider in researching preventative measures and interventions for oral disease are the biofilms created by microbiota. Biofilms are the extracellular matrices formed by colonies of oral microbes on the hard surfaces of dentition (88). These biofilms adhere to the tooth surface and encapsulate microbiota within a protective layer of secreted polymers and allow microbes to resist environmental changes (88). Microbial communities are also able to alter biofilm phenotypes in response to change via gene expression patterns (88). Because of this, oral microbial biofilms are notoriously capable of resisting removal and antibiotic or antimicrobial treatment efforts (88, 89). Thus, biofilms are likely to play a significant role in transplant success or antimicrobial product efficacy, and could potentially play a role in NPD development and treatment response.

Lastly, oral microbiomes can also be modulated by shifting one’s lifeway or diet, as discussed above, or potentially by changing one’s environmental exposures. Research is increasingly linking human health and the microorganism in the body to the environments they live in, both through exposure to pollutants and chemicals, but also diverse sets of environmental microbes. A One Health perspective is useful in these contexts, which posits that our health is reliant on the external health of our environments and ecosystems. Using a One Health approach could be valuable to inform health policies aimed at targeting oral and mental health and examining how exposure to environmental microbes may influence oral health. There is precedence of the introduction of environmental microorganisms into the mouth, both via dietary food and water sources (90), although limited research exists in this area. Nevertheless, environmental exposures may play a role in how we think about modulating oral microbiota to address systemic health concerns. For example, city planning for urban areas to include greater diversity of soil microbes through incorporation of natural “green spaces” could help to increase population exposure to beneficial microbes (91), as exposure to these environmental microbes may play key roles in the treatment of NPDs. Specifically, exposure to the soil bacterium *Mycobacterium vaccae* has been shown to have an anxiolytic effect on the host, as the host’s immune response releases anti-inflammatory cytokines that have positive effects on reducing inflammation in the body and brain—a major component in anxiety and depression (92, 93). However, mechanisms that are facilitated via the oral microbiome have not yet been identified. Nevertheless, implementing social policies that provide health, environmental exposures (e.g., requiring that children have time to engage with these spaces daily during school), could further serve to ensure that people are able to consistently access and benefit places with diverse environmental microbes (91).

Microbiome research on NPDs is still in its infancy, but what has been published regarding the OMBA suggests that it may play a critical role in mental and physical health. Already, there are efforts to apply these novel findings in creating treatments for a range of NPDs and NCDs. However, there are still a large number of unknown factors which contribute to the OMBA in healthy and diseased states. As such, when it comes to regulatory policies regarding new oral products, a stance based on precaution would be advisable until further research is able to establish what risks to consumer health they may pose.

## Conclusion

Oral microbiota likely play key roles in the development, progression, and symptoms of NPDs via the OMBA. An interdisciplinary approach to studying the OMBA can help identify unique factors that influence the oral microbiome and potentially mental health outcomes at individual, community, and population levels. Such research can connect the OMBA to real-world outcomes, revealing specific patterns occurring in the microbiome in responses to changing environments and identify populations that may be more vulnerable to NPDs. Including multidisciplinary approaches that address the OMBA may also lead to better identification, prevention, and treatment of NPDs. By acknowledging and addressing the OMBA and the sociocultural and behavioral factors that shape oral microbiota, we can create solutions and implement policies that better target and prevent oral disease and NPDs.

## Author Contributions

GB researched and wrote the review. LW edited the manuscript. Both authors contributed to the article and approved the submitted version.

## Conflict of Interest

The authors declare that the research was conducted in the absence of any commercial or financial relationships that could be construed as a potential conflict of interest.

## Publisher’s Note

All claims expressed in this article are solely those of the authors and do not necessarily represent those of their affiliated organizations, or those of the publisher, the editors and the reviewers. Any product that may be evaluated in this article, or claim that may be made by its manufacturer, is not guaranteed or endorsed by the publisher.
